# Relationship between body composition, inflammation and lung function in overweight and obese asthma

**DOI:** 10.1186/1465-9921-13-10

**Published:** 2012-02-01

**Authors:** Hayley A Scott, Peter G Gibson, Manohar L Garg, Jeffrey J Pretto, Philip J Morgan, Robin Callister, Lisa G Wood

**Affiliations:** 1 Respiratory and Sleep Medicine, Hunter Medical Research Institute, John Hunter Hospital, Newcastle, NSW Australia 2305; 2 School of Medicine and Public Health, The University of Newcastle, NSW Australia 2308; 3 Nutraceuticals Research Group, School of Biomedical Sciences and Pharmacy, The University of Newcastle, NSW Australia 2308; 4 School of Education, The University of Newcastle, NSW Australia, 2308

**Keywords:** adipose tissue, dual energy x-ray absorptiometry, leptin, lung volume measurements, neutrophil, physiology

## Abstract

**Background:**

The obese-asthma phenotype is not well defined. The aim of this study was to examine both mechanical and inflammatory influences, by comparing lung function with body composition and airway inflammation in overweight and obese asthma.

**Methods:**

Overweight and obese (BMI 28-40 kg/m^2^) adults with asthma (n = 44) completed lung function assessment and underwent full-body dual energy x-ray absorptiometry. Venous blood samples and induced sputum were analysed for inflammatory markers.

**Results:**

In females, android and thoracic fat tissue and total body lean tissue were inversely correlated with expiratory reserve volume (ERV). Conversely in males, fat tissue was not correlated with lung function, however there was a positive association between android and thoracic lean tissue and ERV. Lower body (gynoid and leg) lean tissue was positively associated with sputum %neutrophils in females, while leptin was positively associated with android and thoracic fat tissue in males.

**Conclusions:**

This study suggests that both body composition and inflammation independently affect lung function, with distinct differences between males and females. Lean tissue exacerbates the obese-asthma phenotype in females and the mechanism responsible for this finding warrants further investigation.

## Background

Obesity and asthma are associated conditions, with obese asthmatics experiencing more severe asthma symptoms, reduced lung function and poorer asthma-related quality of life, compared to asthmatics of a healthy weight [1,2]. Although these clinical characteristics are well described, the mechanisms responsible are not understood. The most recent evidence suggests that the obese-asthma phenotype has both mechanical [3] and inflammatory [4,5] influences, and that these differ between males and females. Excess adipose tissue exerts a mechanical effect on the lungs, whereby fat tissue within the android (abdominal) region reduces the capacity of the diaphragm to shift downward thereby limiting lung inflation [3]. Fat tissue in the thoracic region reduces chest cavity volume and diminishes chest wall movement [3]. Body composition and fat distribution differ between males and females, which may account for some of the sex differences observed in lung mechanics [6].

The effect of obesity-induced inflammation has also been investigated in asthma and it appears to be sexually dimorphic in nature. We recently reported an increase in neutrophilic airway inflammation in obese asthmatic females compared with non-obese asthmatic females; an observation that was not apparent in males [4]. In addition, previous authors have cited a relationship between asthma incidence and serum leptin in females but not males; and between serum adiponectin levels and poorer lung function [7].

A number of studies have examined the relationship between BMI and respiratory function impairment, with an association cited by some [8], but not all [9,10] authors. These discrepancies are likely due to BMI being a crude marker of obesity that does not account for either the quantity or distribution of fat and lean tissue. This is a critical issue because both adipose and lean tissue exert contrasting mechanical and inflammatory effects. Regional adiposity estimated by skinfold thickness and waist circumference measurement appears to be more consistently related to lung function impairment, particularly within the android and thoracic regions [9,11-13]. However, the sensitivity and reliability of skinfold thickness measurement has been questioned, particularly in obese subjects [14-16] and neither measure accounts for lean tissue. Dual-energy x-ray absorptiometry (DXA) however provides a reliable and superior measurement of body composition by quantifying regional fat and lean tissue (muscle) mass.

The combined mechanical and inflammatory influences of obesity on respiratory function in asthma are not well understood. Therefore, we sought to examine this association within an asthmatic population. We hypothesised that both android and thoracic fat mass and lean mass directly impacts lung function via mechanical influences. We also hypothesised that body composition affects both systemic inflammation and airway inflammation, altering lung function and asthma status independent of these mechanical influences. The aim of this study was to investigate the relationships between lung function and body composition, systemic inflammation and airway inflammation in overweight and obese males and females with asthma. Further, we sought to examine sex-specific differences in these relationships.

## Methods

### Subjects

Non-smoking overweight and obese (BMI 28-40 kg/m^2^) males (n = 20) and females (n = 24) with asthma were recruited from John Hunter Hospital, NSW, Australia, prior to commencement in a weight loss intervention. Asthma was defined by doctor's diagnosis and airway hyperresponsiveness to hypertonic saline. Medical records were examined for documented history of airway hyperresponsiveness; in instances where this was not recorded, participants underwent hypertonic saline challenge prior to admission into the study. All subjects were classified as stable with no asthma exacerbation, respiratory tract infection or oral corticosteroid use in the preceding four weeks. Subjects underwent skin allergy testing and completed the Asthma Control Questionnaire (ACQ) [17]. This research was approved by the Hunter New England Human Research Ethics Committee and all subjects provided written informed consent.

### Lung Function Tests

Dynamic lung function [forced expiratory volume in one second (FEV_1_) and forced vital capacity (FVC)] was measured using a KoKo spirometer (POS Instrumentation, Inc, Louisville USA), while static lung function [total lung capacity (TLC), functional residual capacity (FRC) and expiratory reserve volume (ERV)] was measured using a computerised plethysmograph system (Vmax Encore, Sensormedics Corp., Yorba Linda, Ca, USA). These measurements were conducted in accordance with ATS/ERS guidelines [18,19].

### Anthropometric Measurements

Body weight was determined using calibrated electronic scales (Nuweigh LOG 842, NWS, Newcastle Australia), while participants wore light clothing and no shoes. Height was measured using a wall-mounted stadiometer. Waist circumference was measured using an inelastic tape (Lufkin W606PM Executive Diameter Tape 2 m × 6 mm, Lufkin USA), at the midpoint of the lowest rib and iliac crest [16]. Both height and waist circumference were measured in duplicate, with measurements within 5 mm for height and 10 mm for waist circumference deemed acceptable. All subjects were classified as weight stable, defined as weight maintenance within 5% for the preceding three months.

A total body scan was performed by DXA (Lunar Prodigy Series, GE Medical Systems, Madison USA). Subjects wore light clothing, no shoes and removed unfixed metal objects. Lean mass and fat mass were measured in grams, with %fat calculated as [fat mass/(lean mass+fat mass) × 100]. The android region was defined as extending from the pelvis to 20% of the distance between the neck and pelvis, with the gynoid region extending from 1.5 times the height of the android region inferior to the pelvis and had a height double that of the android region [20]. The thoracic region was defined as the superior segment of the trunk region bisecting at the most infero-lateral limit of the rib cage [6]. Arm regions passed through the arm sockets, while leg regions passed through the hip sockets [20]. DXA has been shown previously to provide a reliable and accurate measure of both adipose and lean tissue, with a precision error of 1.5% for lean mass and < 1.5% for fat mass [21]. Quality assurance and quality control measures were performed daily in accordance with the manufacturer's instructions.

### Sputum Induction and Analysis

Sputum induction coupled with bronchial provocation using 4.5% hypertonic saline was performed over a standardised 15.5 minutes nebuliser time [22]. A decrease in FEV_1 _≥ 15% from baseline was indicative of airway hyperresponsivensss. Provocation dose (PD15) was calculated and β_2_-agonist (salbutamol 200 μg) was administered [22]. Lower respiratory sputum portions were selected and dispersed using dithiothreitol. Total cell counts and cell viability (trypan blue exclusion) were determined by haemocytometer. Differential cell counts were determined from cytospins, which were prepared, stained (May-Grunwald Geimsa) and counted from 400 non-squamous cells. Absolute cell counts were calculated in a standardised manner. For example, absolute sputum neutrophils were calculated as [(%Neutrophils/100) × Total Cell Count] × 1000, where the total cell count is expressed as × 10^6 ^cells/mL.

### Serum Inflammatory Markers

Venous blood was collected after a 12-hour overnight fast. C-reactive protein (CRP) was measured in heparinised plasma using the CRP Flex^® ^reagent cartridge (Siemens Healthcare Diagnostics Inc., Newark USA) and automatically analysed by the Dimension Vista^® ^System (Siemens Healthcare Diagnostics Inc., Newark USA). The limit of quantitation was 2.9 mg/L. For samples below this limit, additional heparinised plasma was processed using the CRP Extended Range method [23] with results automatically determined using the Dimension^® ^System (Siemens Healthcare Diagnostics Inc., Newark USA). The analytical sensitivity of this method was 0.5 mg/L. A commercial multiplex assay was used to measure serum leptin (Bio-Rad, Hercules CA USA), with a sensitivity of 2.3 pg/mL.

### Statistical Analysis

Data were analysed using a statistical software package (Intercooled Stata version 11.1, Stata Corporation, College Station TX USA) and are reported as median (interquartile range), mean ± standard deviation or % the percentage of subjects with the specified variable. Continuous data were compared by sex using the Kruskal-Wallis test with post-hoc Wilcoxon rank sum testing or ANOVA with post-hoc two sample *t*-testing. Sex-specific associations between lung function and systemic inflammation, airway inflammation and body composition were determined by partial correlations adjusted for height, age and pack years. Non-parametric variables were transformed. Sex-stratified backward and forward stepwise regression was used to verify the independent effects of both central body composition (android and gynoid) and inflammation on ERV and FEV_1_/FVC, until the most parsimonious model was achieved. Due to android and thoracic mass being highly correlated, thoracic lean mass and fat mass were not entered into the regression models. Models including ERV (litres) were adjusted for height and age, and all multivariate models were age-adjusted. *P*-values ≤0.2 entered the multivariate model. *P*-values ≤0.05 were considered statistically significant.

## Results

### Subject Characteristics

Characteristics of the 44 subjects are presented in Table 1. Males and females were of a similar age, however females had a significantly higher BMI (*p *= 0.006) and total and regional %body fat (*p *< 0.001). %Predicted lung function, age of asthma onset and steroid use were similar between the sexes. Males had a higher eosinophil concentration in the sputum and a significantly greater proportion were atopic (Table 1).

**Table 1:** Subject Characteristics.

	Males	Females
N (%)	20 (45.5)	24 (54.5)
Age (years)	40.8 ± 11.4	38.8 ± 14.9
BMI (kg/m^2^)	32.2 ± 2.9	35.1 ± 3.6**
Waist Circumference (cm)	113.0 ± 10.1	107.8 ± 9.6
Total Body Fat (%)	35.2 ± 5.0	50.3 ± 3.8***
Android Fat (%)	46.2 (41.6, 51.5)	55.8 (52.7, 59.9)***
Gynoid Fat (%)	37.0 ± 4.6	55.6 ± 3.9***
Thoracic Fat (%)	35.4 ± 5.1	45.9 ± 4.9***
FEV_1 _%predicted	79.3 ± 19.5	83.3 ± 17.5
FVC %predicted	96.7 (84.0, 98.8)	96.4 (85.8, 107.9)
FEV_1_/FVC	66.5 (58.2, 78.9)	73.7 (67.1, 78.4)
TLC %predicted	98.1 ± 13.6	97.6 ± 12.0
FRC %predicted	101.5 ± 27.5	89.8 ± 19.0
ERV %predicted	112.2 (87.3, 130.4)	110.1 (93.3, 127.4)
Atopy (%)	100.0	78.3*
ICS Dose (μg/day) †	1000 (0, 1250)	1000 (0, 1000)
ICS Use (%)	70.0	70.8
Ever Smokers (%)	35.0	33.3
Pack Years of Ever Smokers	8 (1, 15)	3 (1, 7)
Age at Asthma Diagnosis (Years)	6 (2, 16)	8 (3, 18)
ACQ Score	1.2 ± 0.7	1.4 ± 0.5
GINA Classification (%)		
Intermittent	25.0	12.5
Mild Persistent	20.0	29.2
Moderate Persistent	30.0	45.8
Severe Persistent	25.0	12.5
Systemic Inflammatory Markers		
Leptin (μg/L)	6.2 (4.4, 10.9)	28.4 (19.7, 33.4)***
C-Reactive Protein (mg/L)	1.6 (0.9, 5.0)	4.8 (2.8, 8.3)**
Airway Inflammatory Markers		
Total Cell Count (× 10^6^/mL)	2.6 (2.1, 4.1)	2.0 (1.4, 5.6)
Neutrophils (%)	31.3 ± 19.8	35.5 ± 24.9
Neutrophils (× 10^4^/mL)	752 (321, 1641)	1051 (277, 2709)
Eosinophils (%)	4.3 (2.2, 10.3)	1.3 (0.5, 3.3)*
Eosinophils (× 10^4^/mL)	184 (68, 375)	100 (11, 133)*

Data presented as mean ± standard deviation, median (IQR) or % the percentage of subjects with the specified variable. BMI - body mass index; FEV_1 _- forced expiratory volume in one second; FVC - forced vital capacity; TLC - total lung capacity; FRC - functional residual capacity; ERV - expiratory reserve volume. * *p*≤0.05 versus males; ** *p*≤0.01 versus males; *** *p*≤0.001 versus males; † Beclomethasone equivalents.

### Body Composition and Lung Function

There were strong inverse correlations between waist circumference and static lung function and FVC in females (Table 2). There were also strong inverse correlations between both ERV and FRC versus android fat mass, thoracic fat mass and arm fat mass (Table 2). There was also an inverse association between FEV_1 _and FVC with arm fat mass in females. Of all the body composition measurements, only %fat and total fat mass of the arms correlated with FEV_1 _in females. There was no association between waist circumference or fat mass with lung function in males (Table 2).

**Table 2:** Relationship between body composition and static lung function measurements in males and females with asthma.

	Males			Females		
	
	TLC (L)	FRC (L)	ERV (L)	TLC (L)	FRC (L)	ERV (L)
**Weight (kg)**	0.034	0.157	0.482	-0.354	-0.516*	-0.532*
**Waist Circumference (cm)**	-0.125	0.086	0.494	-0.439*	-0.479*	-0.469*
**Fat Mass (g)**						
Total	-0.424	-0.208	0.257	-0.396	-0.506*	-0.400
Android	-0.434	-0.137	0.263	-0.275	-0.457*	-0.505*
Gynoid	-0.134	-0.132	0.456	-0.087	-0.285	-0.046
Thoracic	-0.378	-0.130	0.319	-0.270	-0.488*	-0.564**
Arms	-0.341	-0.250	0.207	-0.719***	-0.562**	-0.579**
Legs	-0.040	-0.098	0.267	-0.202	-0.299	-0.047
**Lean Mass (g)**						
Total	0.561*	0.409	0.164	-0.157	-0.261	-0.463*
Android	0.566*	0.670**	0.666**	-0.038	-0.142	-0.375
Gynoid	0.383	0.406	0.531*	-0.019	-0.211	-0.360
Thoracic	0.460	0.441	0.540*	-0.057	-0.198	-0.401
Arms	0.497*	0.498*	0.040	-0.501*	-0.367	-0.441*
Legs	0.523*	0.227	-0.157	-0.195	-0.257	-0.323
**%Fat**						
Total	-0.563*	-0.322	0.180	-0.298	-0.319	-0.070
Android	-0.691**	-0.419	-0.007	-0.332	-0.473*	-0.321
Gynoid	-0.257	-0.280	0.331	-0.077	-0.149	0.141
Thoracic	-0.610*	-0.337	0.079	-0.265	-0.401	-0.350
Arms	-0.445	-0.250	0.181	-0.595**	-0.484*	-0.460*
Legs	-0.201	-0.177	0.325	-0.090	-0.138	0.130

Partial correlations are adjusted for height, age and pack years. TLC - logarithmic total lung capacity; FRC - logarithmic functional residual capacity; ERV - expiratory reserve volume. * *p*≤0.05; ** *p*≤0.01; *** *p*≤0.001

Although fat mass was not significantly associated with lung function in males, lean mass had a significant positive relationship. Android lean mass was positively associated with static lung function, with ERV also positively associated with thoracic and gynoid lean mass (Table 2). These associations did not reach significance for dynamic lung function measures (Table 3). Conversely, a negative correlation between lean mass and lung function was observed in females. Total body lean mass was inversely associated with ERV, while arm lean mass was inversely associated with ERV, TLC and FVC (Tables 2 and 3).

**Table 3:** Relationship between body composition and dynamic lung function measurements in males and females with asthma.

	Males (n = 19)			Females (n = 24)		
	
	FEV_1 _(L)	FVC (L)	FEV_1_/FVC (L)	FEV_1 _(L)	FVC (L)	FEV_1_/FVC (L)
**Weight (kg)**	0.035	0.030	0.057	-0.239	-0.402	0.140
**Waist Circumference (cm)**	-0.007	-0.077	0.102	-0.399	-0.569**	0.019
**Fat Mass (g)**						
Total	0.028	-0.101	0.164	-0.192	-0.349	0.181
Android	-0.018	-0.187	0.162	-0.155	-0.330	0.163
Gynoid	0.081	-0.028	0.145	0.148	0.090	0.255
Thoracic	-0.014	-0.128	0.119	-0.119	-0.323	0.219
Arms	-0.092	-0.172	0.038	-0.535*	-0.753***	0.046
Legs	0.074	0.096	0.119	-0.097	-0.123	0.076
**Lean Mass (g)**						
Total	0.296	0.461	0.059	-0.255	-0.333	-0.048
Android	-0.165	-0.098	-0.361	-0.229	-0.259	-0.100
Gynoid	0.124	0.244	0.026	-0.063	-0.128	0.050
Thoracic	0.169	0.397	-0.067	-0.186	-0.271	-0.011
Arms	-0.283	-0.137	-0.342	-0.341	-0.501*	0.011
Legs	0.377	0.498	0.143	-0.314	-0.350	-0.130
**%Fat**						
Total	-0.099	-0.270	0.122	0.000	-0.101	0.227
Android	0.051	-0.246	0.333	0.016	-0.191	0.318
Gynoid	0.003	-0.077	0.115	0.171	0.162	0.210
Thoracic	-0.134	-0.368	0.147	0.035	-0.140	0.278
Arms	0.122	-0.002	0.224	-0.481*	-0.651***	0.036
Legs	-0.071	-0.076	0.006	0.061	0.082	0.118

Partial correlations are adjusted for height, age and pack years. FEV_1 _- Forced expiratory volume in one second; FVC - forced vital capacity. * *p*≤0.05; ** *p*≤0.01; *** *p*≤0.001.

### Body Composition, Systemic and Airway Inflammation

Fat mass, weight and waist circumference were positively associated with serum leptin in males, while plasma CRP was negatively associated with leg lean mass (Table 4). In females, both sputum %neutrophils and absolute neutrophil count were positively associated with lower body (gynoid and leg) lean mass (Table 5). Interestingly, no association was observed between neutrophils and fat mass in males (Table 5). Also there was also no significant association between systemic inflammation and fat mass in females (Table 5). However, gynoid fat mass was associated with a lower concentration of airway eosinophils and a lower provocation fall after hypertonic saline challenge (dose response slope) in females (Table 5).

**Table 4:** Relationship between body composition and systemic inflammation (leptin, CRP), airway inflammation and airway hyperresponsiveness in males.

	Leptin (pg/mL)	CRP (pg/mL)	Neutrophils (%)	Neutrophils (× 10^4^/mL)	Eosinophils (%)	Eosinophils (× 10^4^/mL)	**Dose Response Slope **†
**Weight (kg)**	0.533*	0.139	-0.200	-0.145	0.349	0.090	-0.198
**Waist Circumference (cm)**	0.608*	0.217	-0.177	-0.097	0.433	0.118	-0.220
**Fat Mass (g)**							
Total	0.749**	0.406	-0.160	-0.039	0.382	0.107	-0.192
Android	0.726**	0.409	-0.225	-0.038	0.380	0.052	-0.272
Gynoid	0.499	0.232	-0.106	-0.230	0.294	-0.053	-0.213
Thoracic	0.675**	0.400	-0.232	-0.195	0.434	0.059	-0.305
Arms	0.597*	0.444	-0.017	-0.064	0.538*	0.228	-0.162
Legs	0.402	0.235	-0.096	-0.060	0.239	0.099	-0.077
**Lean Mass (g)**							
Total	-0.120	-0.407	0.033	0.244	-0.062	0.225	-0.120
Android	0.333	0.005	-0.301	-0.029	0.344	0.379	0.242
Gynoid	0.084	-0.256	-0.242	-0.225	0.405	0.218	-0.223
Thoracic	0.051	-0.173	-0.258	-0.158	0.298	0.261	-0.206
Arms	-0.462	-0.136	-0.252	-0.186	-0.110	-0.020	0.027
Legs	-0.344	-0.604*	0.186	0.265	-0.395	0.005	-0.078
**%Fat**							
Total	0.735**	0.502*	-0.220	-0.166	0.465	0.071	-0.137
Android	0.634*	0.407	-0.134	-0.090	0.267	-0.095	-0.395
Gynoid	0.507	0.346	-0.100	-0.255	0.311	-0.088	-0.109
Thoracic	0.681**	0.511*	-0.182	-0.182	0.416	-0.011	-0.248
Arms	0.658**	0.377	0.112	0.027	0.354	0.093	-0.246
Legs	0.498	0.439	-0.180	-0.192	0.418	0.103	-0.027

CRP - C-reactive protein. Partial correlations are adjusted for height, age and pack years. * *p*≤0.05; ** *p*≤0.01; *** *p*≤0.001. † Provocation fall (%) ÷ provocation dose of 4.5% hypertonic saline (mL), after hypertonic saline challenge.

**Table 5:** Relationship between body composition and systemic inflammation (leptin, CRP), airway inflammation and airway hyperresponsiveness in females.

	Leptin (pg/mL)	CRP (pg/mL)	Neutrophils (%)	Neutrophils (× 10^4^/mL)	Eosinophils (%)	Eosinophils (× 10^4^/mL)	**Dose Response Slope **†
**Weight (kg)**	0.391	0.199	0.218	0.263	-0.370	-0.291	-0.196
**Waist Circumference (cm)**	0.349	0.048	0.285	0.306	-0.125	-0.229	-0.045
**Fat Mass (g)**							
Total	0.415	0.211	-0.040	-0.072	-0.506*	-0.385	-0.270
Android	0.260	0.240	0.029	0.068	-0.333	-0.315	-0.113
Gynoid	0.316	0.168	-0.115	0.032	-0.650**	-0.298	-0.480*
Thoracic	0.244	0.156	0.017	0.011	-0.413	-0.459	-0.209
Arms	0.317	0.036	0.026	-0.015	-0.031	-0.209	0.021
Legs	0.315	0.067	0.044	0.199	-0.438	-0.129	-0.362
**Lean Mass (g)**							
Total	0.288	0.225	0.461	0.408	-0.048	-0.109	-0.060
Android	0.205	0.243	0.322	0.279	-0.042	-0.115	-0.122
Gynoid	0.232	0.247	0.544*	0.555*	-0.159	-0.038	-0.125
Thoracic	0.193	0.180	0.323	0.273	-0.149	-0.227	-0.127
Arms	0.277	0.111	0.227	0.171	-0.077	-0.231	0.270
Legs	0.309	0.045	0.709***	0.696**	0.070	0.119	0.087
**%Fat**							
Total	0.216	0.110	-0.474*	-0.349	-0.448	-0.324	-0.296
Android	0.166	0.112	-0.298	-0.205	-0.353	-0.263	-0.050
Gynoid	0.188	0.103	-0.441	-0.337	-0.512*	-0.279	-0.451*
Thoracic	0.082	0.072	-0.306	-0.287	-0.334	-0.340	-0.133
Arms	0.317	-0.015	-0.076	-0.087	0.017	-0.094	-0.146
Legs	0.147	0.095	-0.345	-0.231	-0.445	-0.251	-0.420

CRP - C-reactive protein. Partial correlations are adjusted for height, age and pack years. * *p*≤0.05; ** *p*≤0.01; *** *p*≤0.001. † Provocation fall (%) ÷ provocation dose of 4.5% hypertonic saline (mL), after hypertonic saline challenge.

### Lung Function, Body Composition, Systemic and Airway Inflammation

In a multiple linear regression model, ERV was positively associated with android lean mass in males (Table 6) but not females (Table 7). For every 100 g increase in android lean mass in males, there was a corresponding increase in ERV of 29 mL. Android fat mass was negatively associated with ERV in females (Table 7) but not males (Table 6), whereby every 100 g increase in android fat mass resulted in a decrease in ERV of 20 mL. Inflammation was a more important predictor of dynamic lung function, as there was a negative relationship between FEV_1_/FVC and sputum %eosinophils in males (Table 6) and sputum %neutrophils in females (Table 7). For every 1% increase in sputum %eosinophils in males and 5% increase is sputum %neutrophils in females, there was a corresponding decrease in FEV_1_/FVC of 0.6%.

**Table 6:** Multiple linear regression examining the relationship of body composition, airway inflammation and systemic inflammation, with lung function in males.

ERV (mL)	Unadjusted Model		Final Model	
			*R*-squared = 0.681	*p *< 0.001
**Variable**	**β-Coefficient (95% CI)**	***p-*value**	**β-Coefficient (95% CI)**	***p-*value**

Lean Mass - Android (g)	**0.29 (0.03, 0.55)**	**0.032**	**0.29 (0.03, 0.55)**	**0.032**
Lean Mass - Gynoid (g)	0.3 (-0.1, 0.7)	0.082		
%Sputum Eosinophils	19.67 (-6.24, 45.57)	0.125		
Leptin (pg/mL)	0.025 (-0.002, 0.051)	0.063		
Age (years)	**-28.2 (-51.2, -5.2)**	**0.019**	**-40.3 (-58.0, -22.7)**	**< 0.001**
Height (cm)	12.6 (-18.3, 43.5)	0.400	-9.5 (-40.8, 21.8)	0.529

**FEV_1_/FVC**	**Unadjusted Model**		**Final Model**	
			***R*****-squared = 0.510**	***p *****<0.001**

**Variable**	**β-Coefficient (95% CI)**	***p-*value**	**β-Coefficient (95% CI)**	***p-*value**

Lean Mass - Android (g)	**-0.006 (-0.012, -0.002)**	**0.014**	**-0.007 (-0.012, -0.002)**	**0.014**
%Sputum Neutrophils	0.1 (-0.1, 0.3)	0.149		
%Sputum Eosinophils	**-0.56 (-1.09, -0.03)**	**0.039**	**-0.6 (-1.0, -0.2)**	**0.009**
CRP (pg/mL)	**-2.3 (-4.0, -0.6)**	**0.010**		
Age (years)	-0.3 (-0.7, 0.2)	0.225	-0.2 (-0.6, 0.1)	0.141

ERV - expiratory reserve volume; FEV_1 _- forced expiratory volume in one second; FVC - forced vital capacity; CRP - C-reactive protein.

**Table 7:** Multiple linear regression examining the relationship of body composition, airway inflammation and systemic inflammation, with lung function in females.

ERV (mL)	Unadjusted Model		Final Model	
			*R*-squared = 0.569	*p *< 0.001
**Variable**	**β-Coefficient (95% CI)**	***p-*value**	**β-Coefficient (95% CI)**	***p-*value**

Fat Mass - Android (g)	**-0.20 (-0.39, -0.01)**	**0.037**	**-0.20 (-0.39, -0.01)**	**0.037**
Lean Mass - Android (g)	-0.24 (-0.58, 0.11)	0.173		
Age (years)	**-20.7 (-30.7, -10.7)**	**< 0.001**	**-16.0 (-25.0, -7.0)**	**0.001**
Height (cm)	11.4 (-29.0, 51.8)	0.564	-29.5 (-15.6, 74.5)	0.188
				

**FEV_1_/FVC**	**Unadjusted Model**		**Final Model**	

			***R*****-squared=0.264**	***p*****= 0.020**
**Variable**	**β-Coefficient (95% CI)**	***p-*value**	**β-Coefficient (95% CI)**	***p-*value**

%Sputum Neutrophils	**-0.15 (-0.29, -0.05)**	**0.007**	**-0.13 (-0.23, -0.03)**	**0.012**
Age (years)	**-0.3 (-0.5, -0.1)**	**0.010**	-0.1 (-0.3, 0.2)	0.561

ERV - expiratory reserve volume; FEV_1 _- forced expiratory volume in one second; FVC - forced vital capacity.

## Discussion

This study examined the association of lung function with body composition and systemic and airway inflammation, in overweight and obese males and females with asthma. The findings of this study demonstrate a negative mechanical effect of adipose tissue on lung function within both the android and thoracic regions, with this relationship being particularly evident in females. Interestingly, while lean mass within the android and thoracic regions was associated with improved lung function in males, it had a negative or neutral effect in females. Furthermore, the mechanical effect of obesity appears to have a more significant impact on static lung function, whereas both systemic and airway inflammation have greater impact on dynamic lung function. We also found neutrophilic airway inflammation was positively associated with gynoid and leg lean mass but not fat mass in females.

In females, we found that for every 100 g decrease in android fat mass there was a corresponding increase in ERV of 20 mL. This indicates that central fat mass reduction is an important mechanism for improving static lung function in females. However the inflammatory influences we observed indicate that the relationship between respiratory function and obesity in females is more complex. The inflammatory effect of obesity on lung function is occurring via innate immune pathways, as we observed a positive association between sputum neutrophils, and lean mass, as well as lung function. This is in agreement with other authors, who show that it is not airway eosinophilia driving the association between obesity and asthma [24-27]. This also supports our previous study, where we observed a positive association between sputum neutrophils and BMI in asthmatic females [4]. While other authors have reported no significant association between obesity and neutrophilic airway inflammation, they do illustrate a clear trend of increasing %neutrophils as BMI category increases [25-27]. The lack of statistical significance in these studies may possibly be due to sex effects not being examined [25,27], the small sample size enrolled [25,26] and the high variability in the measurement of sputum neutrophils. Interestingly, the positive relationship we observed in this study was between neutrophilic airway inflammation and lean mass, but not fat mass. Sood et al [28] previously observed a positive association between asthma incidence and lean mass in 1422 females who underwent body composition analysis using DXA. The authors found that asthma incidence was more strongly associated with lean mass than fat mass. The authors hypothesised that this may be due to intramyocellular lipid; that is, fat droplets within skeletal muscle that are highly metabolically active [28]. Intramyocellular lipid levels are higher in females and are highly correlated with circulating leptin and adiponectin levels [29,30]. Therefore, although lean tissue itself is not proinflammatory, the presence of elevated intramyocellular lipid within lean tissue may exert proinflammatory effects. Interestingly, the association we observed was between airway neutrophils and lower body lean tissue. This observation suggests that the relationship may be mediated by leptin. Leptin is secreted 2-3 fold higher from subcutaneous compared with visceral adipose tissue [31] and increases activation of neutrophils *via *TNF-α [32]. This may explain the negative association we observed between respiratory function and lean tissue, the positive association between sputum neutrophils and lower body lean tissue and the positive trend between leptin and lower body lean tissue in females. DXA scanning cannot distinguish intramyocellular lipid from lean tissue, hence additional analysis would be required to test this hypothesis. However, examination of intramyocellular lipids may provide further insight into the obese-asthma association.

In contrast, we observed a positive association between central (android and thoracic) lean mass and static lung function in males. A number of DXA studies have cited a similar relationship in healthy subjects [6,33], with loss of lean mass an important predictor of lung function decline in the elderly [12]. We found that for every 100 g increase in android lean mass, there was a corresponding increase in ERV of 29 mL. This finding suggests an important role for central lean mass in preserving static lung function in males. Surprisingly we did not observe significant associations between fat mass and lung function in males, nor was fat mass a significant predictor of lung function in either of the regression models. This is in contrast to females, for whom fat mass was a considerable predictor of reduced lung function. This supports previous research by Sutherland et al [6] who also observed a greater effect of adipose tissue on the lung function of females compared with males. In contrast to the female subjects, there was no association between neutrophilic airway inflammation and body composition or lung function in males. We did, however, observe a weak positive association between sputum eosinophils and fat mass in males, while sputum eosinophils were a negative predictor of FEV_1_/FVC. This result has not been previously described and further examination of the role of eosinophilic airway inflammation in overweight and obese males may be warranted.

Simple anthropometric measurements such as body weight or waist circumference alone do not accurately describe body composition. We did not find body weight or waist circumference to be significantly related to dynamic or static lung function in males. We propose that this may be due to the opposing effects that lean mass and fat mass have on lung function and the fact that body weight and waist measurement cannot discriminate between these two tissue types. In females, however, both body weight and waist circumference were negatively associated with static lung function. These findings suggest that if lung function prediction equations were to be adjusted for obesity, waist circumference would be a suitable measure in females but not males. This poses a problem in males, as more accurate measures of body composition (i.e. those that quantify lean mass) are not practical within the clinical setting.

A limitation of this trial is the limited sample size and its cross-sectional nature which does not allow for the establishment of causality. However, this study has generated some novel findings that extend existing knowledge in the field and suggest an interesting direction for future research. Another limitation is that DXA is not able to differentiate between visceral and subcutaneous fat, nor can it distinguish intramyocellular lipid from lean tissue. More hazardous and expensive investigational techniques such as computerised tomography (CT) and magnetic resonance imaging (MRI) would be required to evaluate the importance of these measures of fat distribution, however would be advantageous for future trials. Finally, all subjects were within a limited BMI range (28-40 kg/m^2^) and as such these results cannot necessarily be generalised to all individuals.

## Conclusions

In conclusion, our data suggests that body composition and systemic and airway inflammation independently alter respiratory function and that these influences are sexually dimorphic in nature. We found android and upper body adiposity to be negatively associated with lung function in females, whilst lean mass in the android region was an important positive predictor of lung function in males. Our data also indicates that lean tissue is positively associated with neutrophilic airway inflammation in females, however the mechanism driving this association is uncertain. Lean tissue should be examined in depth to elucidate how this tissue may be driving neutrophilic airway inflammation and worsening lung function in females with asthma. This study has documented an interplay between distribution of lean and fat masses, airway and systemic inflammation and lung function in overweight and obese asthma. Our findings suggest that the worsened lung function known to be associated with the obese-asthma phenotype is multifactorial in origin, involving an interplay between distribution of lean and fat masses. The findings of this study also suggest that the obese-asthma phenotype is sexually dimorphic in nature and therefore sex differences must be considered in future trials. Future studies should examine whether age of asthma onset modifies these associations. Additional studies are needed to establish the clinical relevance of these findings, to assist in the management of overweight and obese asthma.

## Abbreviations

ACQ: Asthma Control Questionnaire; BMI: body mass index; CRP: C-reactive protein; CT: computerised tomography; DXA: dual-energy x-ray absorptiometry

ERV: expiratory reserve volume; FEV_1 _- forced expiratory volume in 1 second; FRC: functional residual capacity; FVC: forced vital capacity; MRI: magnetic resonance imaging; TLC: total lung capacity

## Competing interests

PG has participated in the development of educational material and as a speaker in educational symposia sponsored by GlaxoSmithKline, Pharmaxis, Novartis, AstraZeneca, and BoerhingerIngelheim.

## Authors' contributions

HS participated in the design of the study, conducted participant visits, analysed and interpreted the data, and drafted the manuscript. PG participated in the design of the study and helped to draft the manuscript. MG participated in the design of the study and assisted in interpretation of the data and drafting the manuscript. JP assisted with acquisition and interpretation of the data and helped to draft the manuscript. PM participated in the design of the study. RC participated in the design of the study. LW participated in the design and coordination of the study, assisted in interpretation of the data and helped to draft the manuscript. All authors have read and approved the final manuscript.
